# A case of lactic acidosis complicating assessment and management of asthma

**DOI:** 10.1186/1755-7682-1-3

**Published:** 2008-04-15

**Authors:** Tonny V Veenith, Abigail Pearce

**Affiliations:** 1 Specialist Registrar, Department of Anaesthetics, Addenbrookes Hospital, Cambridge, UK; 2 Foundation House Officer Year 2, Neurosciences Critical Care Unit, Addenbrookes Hospital, Cambridge, UK

## Abstract

**Introduction:**

Lactic acidosis often occurs in severely unwell patients presenting to Accident and Emergency. It is commonly associated with either hypoxia or decreased tissue perfusion secondary due to cardiovascular collapse or sepsis.

**Case presentation:**

We present a case of severe lactic acidosis in the presence of normal tissue perfusion and oxygenation in a 31-year-old patient with poorly-controlled asthma. Acidosis promptly reversed on discontinuation of inhaled beta-agonists.

**Conclusion:**

Lactic acidosis secondary to inhaled beta-agonist administration may be a common scenario which can be misinterpreted very easily and can confuse the clinical picture. Further studies will be needed to establish the exact aetiology of this lactic acid production.

## Introduction

Lactic acidosis is diagnosed by a metabolic acidosis, associated with an elevated serum lactate concentration. It often occurs in severely unwell patients presenting to Accident and Emergency, and is commonly associated with either hypoxia or decreased tissue perfusion secondary due to cardiovascular collapse or sepsis. Many drugs may also result in an increase in serum lactate, including Salbutamol.

## Case presentation

Ms XF is a 31 year old Caucasian female who has a diagnosis of uncontrolled asthma which has required repeated admissions over approximately ten years [1]. She is otherwise well with no significant co-morbidities.

Her usual medications are inhaled Salbutamol, Fluticasone accuhaler, nebulised Salbutamol, Salmeterol, Monteleucast 10 mg, Theophylline 300 mg bd, Vitamin D3 and Lansoprazole.

The patient was admitted with a history of cough with green sputum, wheezing and shortness of breath over three days. Initially she treated herself with Salbutamol inhalers and nebulised bronchodilators without improvement. She continued to deteriorate and in A&E she was treated with "back to back" salbutamol nebulisers and transferred to ITU. On admission she had no evidence of hypoxia, infection and hypovolemia as evidenced by clinical examination along with invasive monitoring and investigations such as blood gases, FBC, CRP, and CXR.

In ITU she complained of worsening breathlessness, despite an objective improvement in peak expiratory flow rate and wheeze. Her arterial blood gases at that time showed a compensated metabolic acidosis with high lactate. We excluded all common causes of a metabolic acidosis in this clinical setting including hypoxia, hypovolemia and sepsis. We suspected that the lactic acidosis may have been secondary to nebulised salbutamol, and consequently reduced the dosing interval. This resulted in a reduction in the serum lactic acid level. When the nebulised salbutamol was subsequently stopped the lactic acidosis promptly reversed (Table 1). The patient was transferred to the ward and discharged home uneventfully.

**Table 1:** Arterial blood gas analysis before and after stopping salbutamol. The patient's clinical condition and blood gases improved dramatically after cessation of salbutamol. ABGs after 11 hours and 48 hours post-withdrawal of salbutamol are shown.

Time/Date	pH	PaCO2	PaO2	HCO_3_	BE	Lactate	PEFR	Symptoms/Treatment
11/1/06 21.20 hrs	7.39	3.03 KPa	20.5 KPa	13.6 mmol/l	-11 mmol/l	14.6 mmol/l	230 L/min	Minimal wheeze, SOB^+ ^***Reduced dose of Salbutamol***
11/1/06 24.00 hrs	7.32	3.01 KPa	18.6 KPa	11.7 mmol/l	-14 mmol/l	11.06 mmol/l	240 L/min	No wheeze, hyperventilation^+^
12/1/06 3.00 hrs	7.28	2.29 KPa	11.4 KPa	8.3 mmol/l	-19 mmol/l	15.2 mmol/l	230 L/min	SOB^+++^, hyperventilation
12/1/06 7.00 hrs	7.3	2.13 KPa	13.8 KPa	8.0 mmol/l	-18 mmol/l	10.09 mmol/l	300 L/min	Minimal wheeze ***Nebulised salbutamol discontinued***
12/1/06 23.00 hrs	7.43	3.46 KPa	12.3 KPa	17.6 mmol/l	-7 mmol/l	6.3 mmol/l	300 L/min	No wheeze Continued improvement
14/1/06 10.00 hrs	7.53	3.39 KPa	9.6 KPa	21.0 mmol/l	-2 mmol/l	1.1 mmol/l	320 L/min	Discharged to ward.

In this particular patient, salbutamol and its resultant metabolic acidosis caused us difficulty in assessment and management of her asthma.

## Discussion

Salbutamol is a beta agonist associated with a multitude of systemic side effects. One of the least recognised side effects of salbutamol with clinical consequence is lactic acidosis. Lactic acidosis is commonly associated with either hypoxia or decreased tissue perfusion either due to cardiovascular collapse or sepsis [2]. There are many views regarding the pathogenesis of lactic acidosis in asthma; the most accepted one being due to fatiguing respiratory muscles [3]. Another accepted explanation could be due to effects of ischemia and hypoxia on liver which was unlikely in the above case as this patient had normal liver function [4].

In healthy volunteers salbutamol will increase the oxygen consumption by increasing metabolic rate and serum lactate. This will affect patients with severe asthma, who already limited ventilatory reserve, by increasing their symptoms [5]. In pregnant patients the lactate levels increase when given Ritodrine (beta agonist) for tocolysis [6]. An increase in serum lactate has also been documented paediatric patients, followed by administration of nebulised salbutamol [7]. This resolved after the discontinuation of the drug. Lactic acidosis is much more prominent in intravenous salbutamol than nebulised salbutamol, so intravenous salbutamol should not be routinely prescribed [8].

## Conclusion

In this patient, resolution of wheezing was accomplished by intensive treatment with salbutamol but at the cost of lactic acidosis. This was important because acidosis by itself can result in hyperventilation and a sense of dyspnoea which could be easily mistaken for failure to respond to the treatment, as this particular case exemplifies. Lactic acidosis secondary to inhaled beta-agonist administration may be a common scenario which can be misinterpreted very easily and can confuse the clinical picture. Further studies will be needed to establish the exact aetiology of this lactic acid production.

## List of abbreviations

FBC – Full Blood count, CRP – C Reactive Protein, CXR – Chest X-ray, ABG – Arterial Blood Gases, A&E – Accident and Emergency, ITU – Intensive Treatment Unit, PEFR – Peak expiratory flow rate, ECG – Electrocardiogram.

## Competing interests

The author(s) declare that they have no competing interests.

## Authors' contributions

Both author(s) are involved in writing this case report.
